# Training programs in blood diseases: Mayo Clinic Rochester Hematology–Oncology Fellowship Program

**DOI:** 10.1002/ajh.21714

**Published:** 2010-03-17

**Authors:** Cheng E Chee, Tow S Tan, Timothy J Moynihan, Alexandra P Wolanskyj

**Affiliations:** 1 Department of Oncology, Mayo ClinicRochester, Minnesota; 2 Department of Internal Medicine, Division of Hematology, Mayo ClinicRochester, Minnesota

## Historical Perspectives

The Hematology fellowship program at Mayo Clinic Rochester began in 1965 followed by the Oncology fellowship program in 1973. The two programs later merged in 1990 and since that time, 115 fellows have completed training (Table I). Mayo Clinic Jacksonville followed shortly thereafter in 1996 and most recently, Mayo Clinic Scottsdale started its program in 2006. Mayo Clinic is known for excellence in its three shields: clinical practice, education, and research, all of which are emphasized during the training program. Indeed, our fellowship combines well-rounded clinical training with opportunities to work with world-class faculty in a collegial environment. We believe that the 4-year fully funded fellowship program comprehensively prepares one for a career in either academic medicine or group practice. The well-balanced program offers a multidisciplinary approach to patients with hematologic and oncologic disorders, clinical or basic science research and opportunities to teach Mayo Medical School students and internal medicine residents. Clinical Practice

**TABLE I:** Demographics of the Mayo Clinic Rochester Hematology–Oncology Fellowship Program

Program features	Details
Total no. of fellows at present time	29
Outcomes of graduating fellows in last 10 years	*N* = 66
Academic institution	59% (39/66)
Academic-affiliated practice^a^	20% (13/66)
Private practice	21% (14/66)
Peer-reviewed publications by current fellows (per fellow)	135 (mean: 4.7; range 0–25)
Peer-reviewed publications by fellows during fellowship in last 10 years (per fellow)	297 (mean: 4.5; range: 0–32)
No. of first year positions for 2011	9
No. of Hematology/Oncology hospital beds	67
Inpatient admissions (2009)	Hematology: 1514; Oncology: 1441
Outpatient new patient visits (2009)	Hematology: 4943; Oncology: 5924
Cancer discharges^b^	4,785 (ranked 3rd based on US News and World Report on Best Hospitals)
No. of faculty members	Hematology: 45; Oncology: 37

a Defined as a group practice that conducts independent clinical trials/research and/or are affiliated with a residency program or medical school.

b All Medicare inpatients who received medical or surgical care for a cancer diagnosis in 2005–2007.

**Figure 1:**
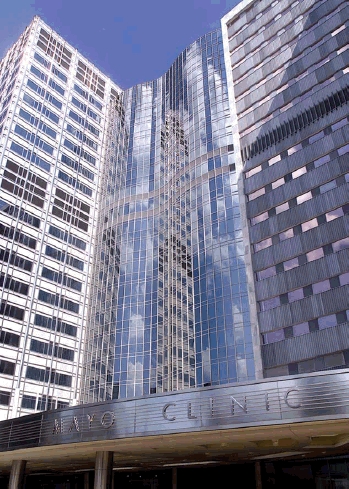
Gonda building in downtown Rochester where the outpatient practice is located. (Reproduced with permission from Mayo Foundation for medical education and research.)

Mayo Clinic is a major national and international referral center whereby a wide variety of oncologic and hematologic malignancies and benign hematologic disorders, are seen by fellows during training under the supervision of recognized experts, enhancing the educational experience and providing patients with the best clinical care. Fellows also provide primary oncologic care for patients with common hematologic and oncologic diseases as Mayo Clinic serves as the primary referral center for the local, tri-state region (Minnesota, Iowa, and Wisconsin). A unique feature of the program is the autonomy and flexibility afforded to our fellows to organize and schedule their own continuity clinic patients. Throughout their training, fellows accumulate patients to their continuity clinic panel, and are looked upon as the patient's primary oncologic or hematologic caregiver, with significant clinical decision responsibilities. This ensures that by the end of fellowship, they are extremely well prepared for clinical practice. Fellows also obtain specialized training during their time spent in our bone marrow transplant program, which performed approximately 280 autologous and 65 allogeneic transplants in 2009. As a regional transplant center, the program continues to expand with the introduction of umbilical cord transplantation in 2009 and an increase in autologous and allogeneic transplants. The outpatient practice is based at the downtown Mayo Clinic facility (Figure 1), while the inpatient Hematology and Oncology services are located at the 794-bed Rochester Methodist Hospital and consult services at the 1265-bed St. Mary's Hospital, all of which have fully computerized and integrated medical records.

## Education

**Figure 2:**
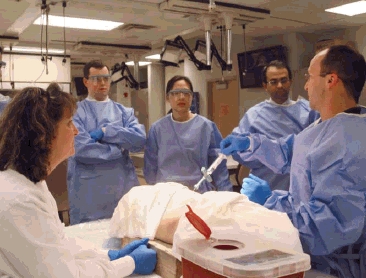
Simulation workshop in the anatomy procedural skills laboratory to teach bone marrow biopsies and aspirates to first-year fellows. (Reproduced with permission from Anatomy Laboratory, Mayo Clinic, Rochester.)

Mayo Clinic's Hematology and Oncology fellowship program utilizes the state-of-the-art multidisciplinary simulation center and anatomy procedure skills laboratory (Figure 2), to train fellows and other medical staff in procedures and problem-solving clinical scenarios. In addition to inpatient and outpatient clinical rotations, fellows rotate through other departments such as Radiation Oncology, Medical Genetics, Palliative Care and a variety of laboratories including, Surgical Pathology, Hematopathology, Cytogenetics, Transfusion Medicine, and Coagulation ensuring comprehensive training. Educational sessions for fellows include a thrice-weekly core lecture series, journal clubs, board review sessions, hematopathology and departmental conferences, medical grand rounds, and annual in-training examinations in Hematology and Oncology to prepare fellows for the board exams. This has proved to be successful as evident from the high-pass rate achieved in the Hematology and Oncology boards.

## Research

Mayo Clinic is a National Cancer Institute (NCI)-designated comprehensive cancer center, and therefore provides a rich menu of research opportunities for fellows to pursue during their 18–22 months of dedicated research time. Multiple programs in respective solid tumor groups, women's cancers, drug development, supportive care, and hematologic malignancies provide fellows with opportunities for either laboratory-based or clinically based research. The institution conducts several weekly cancer-related conferences, thereby allowing fellows to learn of ongoing research efforts and to meet face-to-face with investigators who could serve as potential mentors. There are over 300 active clinical trials open at any one time, and Mayo Clinic serves as the primary research base for the NCI-funded North Central Cancer Treatment Group, the Phase II Consortium (a drug development program), seven different highly competitive SPORE grants (specialized program of research development) in brain, breast, lymphoma, myeloma, ovary, pancreas, and prostate, a community clinical oncology program grant, and a cancer prevention network. Opportunities for basic science, translational, and clinical research are robust on the Mayo Clinic campus. Fellows are involved in writing and development of clinical trials and past and current fellows have been very successful in publishing their research in peer-reviewed journals and presenting abstracts and oral presentations at national and international conferences (Table I). Fellows have the opportunity to obtain a Masters in clinical research from the Mayo School of Graduate Medical Education, during their 4-year program. In addition to the conventional track, a number of our fellows have trained in the Mayo Clinical-Investigator Program, which is designed to prepare leaders in academic medicine. Many of our fellows have successfully obtained appointments in various academic institutions, nationally and internationally, and have received NCI funding and National Institute of Health Career Development awards (K-awards).

Broadly trained and well-mentored, fellows at the Mayo Clinic possess a diverse set of skills which allow them to achieve excellence in all types of academic or community-based practice. Our fellows, exposed to the principles of the founding fathers of the Mayo Clinic, perpetuate their legacy of dedication to clinical care, research, and education.

